# Functional Analysis of Thyroid Peroxidase Gene Mutations Detected in Patients with Thyroid Dyshormonogenesis

**DOI:** 10.1155/2014/390121

**Published:** 2014-04-13

**Authors:** Srikanta Guria, Biswabandhu Bankura, Nisha Balmiki, Arup Kumar Pattanayak, Tapas Kumar Das, Anirban Sinha, Sudipta Chakrabarti, Subhankar Chowdhury, Madhusudan Das

**Affiliations:** ^1^Post Graduate Department of Zoology, Barasat Government College, Barasat, Kolkata 700 124, India; ^2^Department of Zoology, University of Calcutta, 35 Ballygunge Circular Road, Kolkata, West Bengal 700 019, India; ^3^Bagnan Rural Hospital, Bagnan, Howrah, West Bengal 711 303, India; ^4^Department of Endocrinology, Institute of Post Graduate Medical Education & Research, 244 A J C Bose Road, Kolkata 700 020, India; ^5^Institute of Life Sciences, Nalco Square, Chandra Sekharpur, Bhubaneswar 751 023, India

## Abstract

Thyroid peroxidase (TPO) is the key enzyme in the biosynthesis of thyroid hormones. We aimed to identify the spectrum of mutations in the *TPO* gene leading to hypothyroidism in the population of West Bengal to establish the genetic etiology of the disease. 200 hypothyroid patients (case) and their corresponding sex and age matched 200 normal individuals (control) were screened depending on their clinical manifestations. Genomic DNA was isolated from peripheral blood samples and *TPO* gene (Exon 7 to Exon 14) was amplified by PCR. The PCR products were subjected to sequencing to identify mutations. Single nucleotide changes such as Glu 641 Lys, Asp 668 Asn, Thr 725 Pro, Asp 620 Asn, Ser 398 Thr, and Ala 373 Ser were found. Changes in the TPO were assayed *in vitro* to compare mutant and wild-type activities. Five mutants were enzymatically inactive in the guaiacol and iodide assays. This is a strong indication that the mutations are present at crucial positions of the *TPO* gene, resulting in inactivated TPO. The results of this study may help to develop a genetic screening protocol for goiter and hypothyroidism in the population of West Bengal.

## 1. Introduction

TPO is a membrane-bound glycoprotein (102 kDa), found as a dimer [1]. Each monomer consists of 933 amino acid residues and contains a peroxidase domain, three additional extracellular domains, a transmembrane helix, and a short C-terminal intracellular tail [2]. The human* TPO* gene is located on chromosome 2p25 and spans approximately 150 Kb, containing 17 exons [3]. Mutations in* TPO* gene (particularly nonsynonymous cSNPs) can lead to severe defects in thyroid hormone production, due to total iodide organification defects (TIOD) or partial iodide organification defects (PIOD).* TPO* mutations are inherited as autosomal recessive traits [4]. Iodination of salt is the most effective and sustainable long-term public health measure for the prevention and control of iodide organification defects (IDD). But the iodination programme will not be effective if the gene mutations are the cause of dyshormonogenesis. Therefore, it is important to screen the percentage of people having gene mutations and iodine deficiency among the clinically identified thyroid patients.

Screening and identification of mutations in the* TPO* gene of patients with evidence of TIOD and PIOD have been done by several groups in different countries of the world like Argentina [4], Netherlands [6], Japan [7], Portugal [8], and China [9]. The present investigation is aimed at screening the mutations/polymorphisms in* TPO* gene and their effects on the function of* TPO* gene leading to hypothyroidism in the population of West Bengal to establish the genetic etiology of the disease.

## 2. Materials and Methods

200 hypothyroid patients (case) and their corresponding sex and age matched 200 normal individuals (control) were screened depending on their clinical manifestations and detailed familial history from the Institute of Post Graduate Medical Education & Research (IPGME & R), Kolkata. Peripheral blood samples were collected on the basis of prior consent given by patients/normal individuals, families, and parents on behalf of minor children. To the extent possible, complete pedigree samples were obtained which included the proband, siblings, parents, and other blood relatives. Age and sex matched subjects with no goitre, no clinical evidence of hypothyroidism, and normal levels of serum T_3_, free T_4_ (FT_4_), TSH, and anti-TPO antibody were enrolled as normal (control). The experimental protocol was approved by the institutional ethics committee of IPGME & R, Kolkata. Clinical information includes complains like lethargy, cold intolerance, loss of memory, constipation, and weight gain. Personal history includes menarche, last child birth, abortion, cycle duration, and periodicity. Delayed milestones for children like sitting without support, speech, and walking were also documented.

Quantitative sandwich immunoassay kit (Siemens, India) was used to assay serum TSH level. Serum T_3_ or FT_4_ concentrations were determined by radioimmunoassay (RIA). Quantitative measurement of serum TPO antibody was performed using ELISA method. TPO antibody value >60 IU/mL was considered as positive [10]. Radiolabeled iodine was administered to patients and control. Emittance of radioactivity was measured over the thyroid. Potassium perchlorate (a competitive inhibitor of iodide transport into the thyroid) was administered. Emittance of radioactivity was measured over the thyroid and compared to initial result.

The iodine in the urine is measured by a modification of the traditional colorimetric method of Sandell and Kolthoff (1937) [11]. This was done using the ammonium persulfate method as described by Pino et al. (1996) [12]. Urine was digested with ammonium persulfate. The iodine in the urine samples catalyses the reduction of ceric ammonium sulphate (yellow colour) to the cerous form (colourless) in the presence of arsenious acid. The degree of reduction in colour intensity of the yellow ceric ammonium sulphate is proportional to the iodine content in the urine sample.

Peripheral blood samples were collected from the case and control individuals. Genomic DNA was isolated from the blood leucocytes by using QIAamp Blood Kit (QIAGEN, Hilden, Germany). The human* TPO* gene (Exon 7 to Exon 14) was amplified by PCR. PCR was performed in a thermocycler (Applied Biosystems, Model number 9902) using specific primers for each of the exons (Table 1). The reaction mixture (25 *μ*L) contained 40–100 ng of genomic DNA, 1.5 mM MgCl_2_, 100 *μ*M of each dNTP, 0.4 *μ*M of each primer, and 0.5 unit of Taq DNA polymerase (Applied Biosystems). Denaturation at 95°C for 30 seconds, annealing at 55–60°C for 30 seconds, and extension at 72°C for 30 seconds × 44 cycles were performed. The PCR product was analyzed in 2% agarose gel electrophoresis for verifying the size of the PCR product. PCR fragments were purified from agarose gel using Gel Extraction kit (Genei Bangalore).

The PCR products were sequenced by using Big Dye Terminator kit v3.1 (Applied Biosystems) in ABI Prism 377 DNA Sequencer (PE Applied Biosystems). Purified amplicons were used for sequencing reaction and the reaction mixture (10 *μ*L) contained 150 ng DNA, 5 *μ*M primer (either forward or reverse), 0.5 *μ*L BD, and 1.9 *μ*L 5 × BD buffer. Denaturation at 96°C for 10 seconds, annealing at 50–55°C for 10 seconds, and extension at 60°C for 4 min × 25 cycles were performed. 10 *μ*L PCR mix was added with solution I (10 *μ*L mq H_2_O, 2 *μ*L 125 mM EDTA, and pH 8.0) and solution II (50 *μ*L ehanol, 2 *μ*L Na-acetate, and pH 4.6). The mixture was kept in dark for 25 min and centrifuged at room temperature (13000 rpm for 30 min) and sample was dried in speed vac. Then, 12 *μ*L Hi-Di (formamide) was added in sample and stored at dark for 15 min and incubated at 96°C dry bath and we allowed the sample for snap chill in ice. Then, the sample was loaded in DNA Sequencer.

Sequences alignment between sequences of case and control individuals was performed to find the best matching piecewise (local) or global alignments of two query sequences using clustalW programme.

Polymorphism in the* TPO* gene, identified in this study, including nonsynonymous SNPs in the coding region, deletions, and insertions have been assayed* in vitro*. We used Invitrogen GeneArt Site-Directed Mutagenesis System for this purpose, for example, Glu 641 Lys, Asp 668 Asn, Thr 725 Pro, Asp 620 Asn, Ser 398 Thr, and Ala 373 Ser; these changes were incorporated into wild TPO cDNA through this method.

We used pcDNA3.1 (vector) for TPO cDNA cloning. Double restriction enzyme (EcoRI, KpnI) digestions were performed by incubating TPO cDNA and vector molecules with an appropriate amount of restriction enzyme, in its respective buffer as recommended by the supplier, and at the optimal temperature. The cut DNA fragment and vector are covalently joined together by DNA ligase.

Transformation is the method of introducing foreign DNA into bacterial cells. The recombinant plasmids (*TPO* cDNA + plasmid) were amplified in DH5 alpha competent cells. The uptake of recombinant plasmids by DH5 alpha cell was carried out in ice-cold CaCl_2_ (0–5°C) and subsequent heat shock (42°C for about 90 sec). Selection of DH5 alpha competent cells containing recombinant plasmids (TPO cDNA + plasmid) was done by ampicillin treatment. Plasmid was purified by use of the plasmid isolation kit (PureLink Quick Plasmid Miniprep Kit, Invitrogen). We used Invitrogen Lipofectamine Reagent for transfection. COS 7 cells were grown in 3.8 cm dishes in DMEM supplemented with 50 mL/L bovine calf serum and penicillin-streptomycin in 5% CO_2_ atmosphere at 37°C. When the cells reached ~90% confluence, they were transfected with 1 *μ*g of recombinant plasmid DNA (wild-type and mutant) per 3.8 cm dish with Lipofectamine. After incubation for 48 h, the cells were harvested for activity study. Cells were harvested with trypsin EDTA treatment and protein concentration was determined on a 100 *μ*L aliquot using the Bio-Rad protein assay (BioRad, Munchen, Germany). The cells were pelleted and subsequently suspended in 0.1% deoxycholate containing 1% aprotinin and incubated for 10 min at 4°C. The extract was microcentrifuged for 5 min, and the supernatant was removed to measure enzymatic activity using the guaiacol and I_3_^−^ assays. For the guaiacol assay, 50 *μ*L of the supernatant was assayed in a final volume of 750 *μ*L, containing 35 mM guaiacol and 0.5 mM H_2_O_2_ in 0.1 M Tris-HCl, pH 8.6. The absorbance at 470 nm was followed and activity was expressed as ΔA · min⁡^−1^ · mg  protein^−1^. For the I_3_^−^ assay, 25 *μ*L of the supernatant was used in a final volume of 750 *μ*L containing 0.1 M potassium phosphate, pH 7.5, 50 mM potassium iodide, and 0.25 mM H_2_O_2_. The absorbance at 353 nm was followed, and activity was expressed as ΔA · min⁡^−1^ · mg  protein^−1^. Spectrophotometric analysis was done on a Shimadzu UV-200.

Thirty micrograms of the deoxycholate extracted membrane protein fraction containing recombinant TPO and normal control TPO were electrophoresed on a 7.5% SDS-polyacrylamide gel. The gel was blotted onto nitrocellulose using a Bio-Rad Mini Protean 2 system (Bio-Rad Labs, Richmond, CA), followed by incubation with rabbit antihuman TPO antibody (abcam, EPR5379). TPO protein was visualized using ECL Western blotting detection reagent (abcam).

### 2.1. Statistical Methods

*χ* ^2^ and Fisher exact tests were used to test the allelic and genotypic associations of each SNP. Student's* t*-test was used to calculate any statistically significant difference of continuous independent variables like age, TSH, and FT4 within the control and patient groups. All tests were done using GraphPad InStat software (GraphPad InStat software, San Diego, CA). Odds ratio and 95% confidential intervals were also calculated using the same software. A Bonferroni correction was applied for multiple testing.

## 3. Results

The case and control groups were well balanced in terms of age and gender. There is no significant difference between two groups (Table 2). Case individuals exhibited varied clinical manifestations. The associated clinical manifestations of hypothyroidism were goiter (65.5%), lethargy (61%), muscle cramp (55%), loss of memory (51%), hoarseness of voice (40.5%), weight loss (8% in male and 30% in female), constipation (55%), cold intolerance (60%), weight gain (45%) etc.,(Table 3).

**Figure 1:**
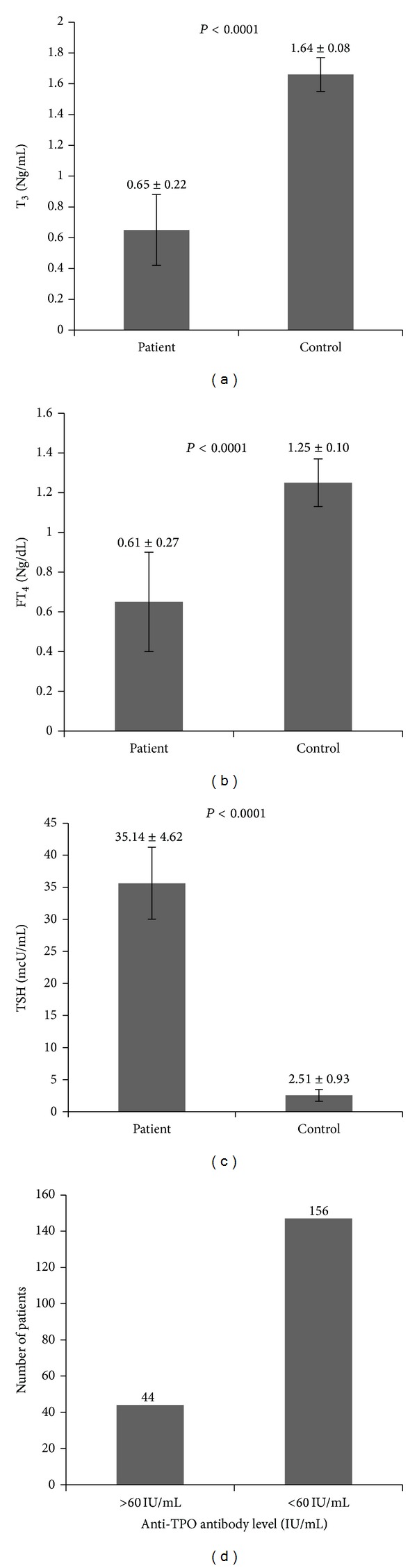
T_3_ and FT_4_ level of patient and control (normal) populations. TSH and anti-TPO antibody level of patient and control (normal) populations.

The TSH level was significantly high and FT_4_ and T_3_ levels were significantly low in case population. In control population average serum TSH level was 2.51 ± 0.93 *μ*U/mL as compared to 35.14 ± 4.62 *μ*U/mL in case population. Similarly, serum FT_4_ level was 1.25 ± 0.10 ng/dL in control population as compared to 0.61 ± 0.27 ng/dL in case population. Serum T_3_ level was 1.64 ± 0.08 ng/dL in control population as compared to 0.65 ± 0.22 ng/dL in case population. 44 patients showed positive TPO antibody level (Figure 1).

The ^123^I uptake as shown by 83 patients at 3 hours was 48.1% ±7.90 and 75.3% ±5.90 of ^123^I was discharged at 60 min after the oral administration of KClO_4_ at a dose of 0.4 g in the perchlorate discharge test, confirming iodide organification defect in the thyroid gland. In our patient population, we found that 192 patients were within the level of 10–20 *μ*g/dL. In overall, 96% of the patients had UIE levels in the ranges of optimal iodine nutrition (10–20 *μ*g/dL) (WHO, UNICEF, and ICCIDD, 2001).

**Figure 2:**
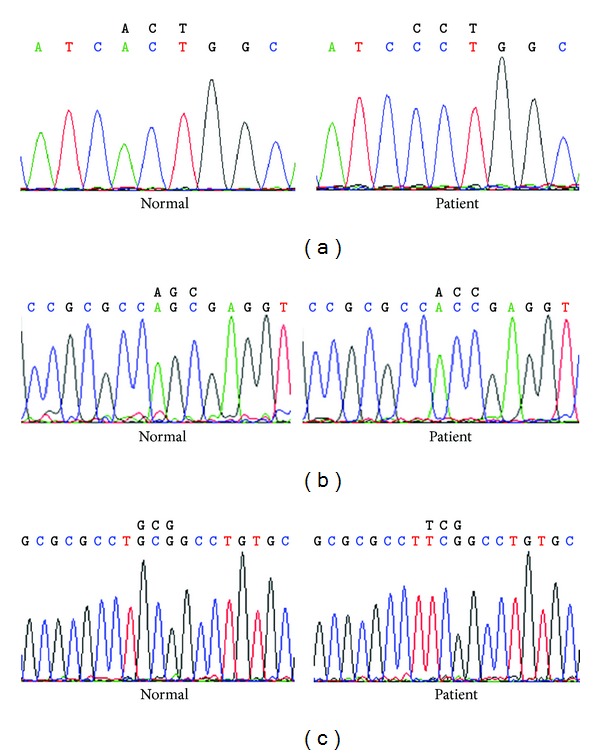
Nucleotide polymorphism in* TPO* gene study population. (a) Nucleotide polymorphism in patient ID-147, Exon11, and ACT > CCT (Thr > Pro). (b) Nucleotide polymorphism in patient ID-6, Exon7, and AGC > ACC (Ser > Thr). (c) Nucleotide polymorphism in patient ID-183, Exon7, and GCG > TCG (Ala > Ser).

Mutation screening of* TPO* gene in both case and control groups, showed six nonsynonymous changes: c. 1117 G>T: Ala 373 Ser, c. 1193G>C: Ser 398 Thr, c. 1858 G>A: Asp 620 Asn, c. 1921 G>A: Glu 641 Lys, c. 2002G>A: Asp668Asn, and c. 2173A>C: Thr725Pro (Figure 2). After the Bonferroni correction for multiple comparisons a strong association was observed between the Asp 620 Asn, Glu 641 Lys, and Thr 725 Pro SNPs and hypothyroidism (Table 4).

To measure wild-type and mutant enzyme activity, both the I^−^ and guaiacol assays were carried out. Wild-type recombinant TPO showed enzymatic activity in both assays. Mutant TPO showed relatively nonenzymatic reaction rate (Table 5).

**Figure 3:**
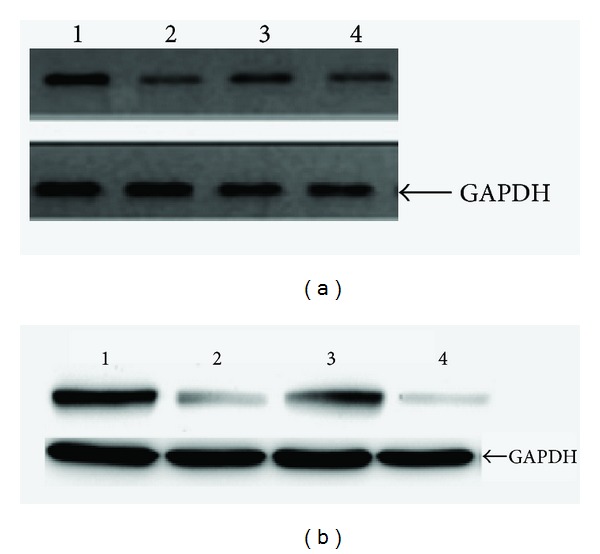
Western blot showing expression of wild-type and mutated recombinant TPO. (a) Lane 1, wild-type recombinant TPO. Lanes 2–4, recombinant TPO containing (2-Glu 641 Lys, 3-Asp 668 Asn, and 4-Thr 725 Pro) changes. In (b) Western blot showing expression of wild-type and mutated recombinant TPO. Lane 1, wild-type recombinant TPO. Lanes 2–4, recombinant TPO containing (2-Asp 620 Asn, 3-Ser 398 Thr, and 4-Ala 373 Ser) changes.

All mutants expressed reduced amount of TPO on the Western blot. In Figure 3(a), Western blot showed expression of wild-type and mutated recombinant TPO. Lane 1 showed wild-type recombinant TPO. Lanes 2–4 showed recombinant TPO containing (2-Glu 641 Lys, 3-Asp 668 Asn, and 4-Thr 725 Pro) changes. In Figure 3(b), Western blot showed expression of wild-type and mutated recombinant TPO. Lane 1 showed wild-type recombinant TPO. Lanes 2–4 showed recombinant TPO Containing (2-Asp 620 Asn, 3-Ser 398 Thr, and 4-Ala 373 Ser) changes.

## 4. Discussion 

The present study reveals clinical analysis of hypothyroidism in screened population. Hypothyroid patients exhibited significant increase in the level of serum TSH than control. As expected, patients showed lower level of T_3_ and FT_4_ as compared to control population. 83 patients showed positive result in the perchlorate discharge test confirming iodide organification defect in the thyroid gland. The urinary iodine excretion (UIE) of 96% patients was within the levels of 10–20 *μ*g/dL which were in the range of optimal iodine nutrition (10–20 *μ*g/dL) (WHO, UNICEF, and ICCIDD 2001). Therefore, UIE levels indicate that iodine deficiency is currently not a public health problem in our screened population.

Our study identified six different SNPs, among these three SNPs (Asp 620 Asn, Glu 641 Lys, and Thr 725 Pro) are significantly associated with hypothyroidism. We also try to find out the association between* TPO* gene polymorphisms and Anti-TPO level in our study subjects and we did not find any association (data not shown). However, several groups in different population like Tehranian population [13] and Iranian population [14] showed association between* TPO* gene polymorphisms and anti-TPO level. Our study needs much larger sample size which may help in better understanding of association between* TPO* gene polymorphisms and anti-TPO level.

Based on the literature, Exons 7–11 encode the catalytic center of the TPO protein (heme binding region) which is crucial for the enzymatic activity [15]. On the other hand, previous findings also indicate that Exons 7–14 harbor mutational hot spots [15]. Thus, mutations in these regions are expected to have major effects on TPO activity resulting in severe organification defect and severe hypothyroidism. In Glu 641 Lys, negative charged amino acid is replaced by positive charged amino acid. As we know, Threonine is the phosphorylation site of the protein which is important for the activation of the protein [16, 17]. Therefore, mutations in this amino acid may change the activity of TPO enzyme which may ultimately reduce the functional efficacy of the enzyme.

We established the effect of these polymorphisms through* in vitro* assay. Some polymorphisms (Glu 641 Lys, Thr 725 Pro, and Asp 620 Asn) were enzymatically inactive in both the guaiacol and iodide assays. They also showed reduced amount of TPO on the Western blot. This is a strong indication that the changes are present at crucial positions of the* TPO *gene, resulting in inactivated TPO.

## 5. Conclusions

Thus, the above changes of amino acids may exert its effect on the structure and functional activity of TPO. Our study tried to detect the genetic etiology of this disease which may further help us to risk categorize for hypothyroidism. This study also helps to develop a genetic screening protocol for hypothyroidism, specifically for the Indian population.

## Figures and Tables

**Table 1:** Primer sequence used to screen the different exons of *TPO* gene.

Exons	Primers	Sequence	Amplicon size (bp)
Exon 7	Forward	5′-CTGGAGCTCTGTGAACAAGAA-3′	433
	Reverse	5′-CCCTGGGAATAGGACAAAGAAA-3′	

Exon 8	Forward	5′-CCCTACGTAACAAACCTGCAC-3′	474
	Reverse	5′-GGCTGTCAAGGAAGATGCTC-3′	

Exon 9	Forward	5′-CGTTGCTTAGAAGGCCTCAG-3′	444
	Reverse	5′-CTTGCAGTGAGCTGAGATCG-3′	

Exon 10	Forward	5′-ACAACCTGACCAGGCTTACG-3′	485
	Reverse	5′-CAGGACTCTGCCCTGCTG-3′	

Exon 11	Forward	5′-CTGCCCTGAGGGTGTAAGG-3′	446
	Reverse	5′-GAGAGGCTGGCAGCACACAG-3′	

Exon 12	Forward	5′-CTATCCCCAGATTGCTCCTG-3′	449
	Reverse	5′-GCTCAGTGAGTGACCACAGC-3′	

Exon 13	Forward	5′-GTGTGCTTCGAGGGTCTCTG-3′	485
	Reverse	5′-CCCTAGACCAGGTGGGATG-3′	

Exon 14	Forward	5′-CCATGTCCAGAGGAAAGGAG-3′	238
	Reverse	5′-CAGACTCAGGCAGGACAACC-3′	

**Table 2:** Study population.

	Case (*n* = 200)	Control (*n* = 200)	*P* value
Sex			
Male	29 (14.5%)	32 (16%)	0.88
Female	171 (85.5%)	168 (84%)	0.80
Age (Mean ± SD)	29.85 ± 19.51	30.38 ± 12.90	

**Table 3:** Clinical manifestations of case at initial presentation.

Clinical manifestations	Case (*n* = 200)	%	Control (*n* = 200)	%	*P* value
*Family history					
Present	93	46.5	6	3	<0.0001
Absent	107	53.5	194	97	
*Goiter					
Present	131	65.5	—	—	
*Weight gain					
Yes	90	45	67	34	0.024
No	110	55	133	66	
Loss of memory					
Yes	102	51	90	45	0.27
No	98	49	110	55	
*Lethargy					
Yes	122	61	91	46	0.002
No	78	39	109	54	
*Muscle cramp					
Yes	110	55	88	44	0.03
No	90	45	112	56	
*Cold intolerance					
Yes	120	60	113	57	0.54
No	80	40	87	43	
*Constipation					
Yes	109	55	80	40	0.005
No	91	45	120	60	

*at the time of diagnosis.

**Table 4:** Allele and Genotype distribution of TPO gene polymorphisms in the study.

SNP	Allele	Allele frequency		Odds ratio (95% CI)	*P* value	Genotype	Case (*n* = 200)	Control (*n* = 200)	Odds ratio (95% CI)	*P* value
		Case	Control							
c. 1117 G>T	G	0.54	0.59	1.23 (0.93–1.62)	0.17	GG	34%	35%	Reference	
Ala 373 Ser	T	0.46	0.41			GT	40%	48.5%	GG versus GT: 0.85 (0.54–1.33)	0.54
						TT	26%	16.5%	GG versus TT: 1.62 (0.94–2.81)	0.11
									GG + GT versus TT: 1.78 (1.09–2.90)	0.028

c. 1193G>C	C	0.73	0.68	0.79 (0.58–1.07)	0.14	CC	57.5%	49%	Reference	
Ser 398 Thr	G	0.27	0.32			GC	30.5%	37.5%	CC versus GC: 0.69 (0.45–1.07)	0.12
						GG	12%	13.5%	CC versus GG: 0.76 (0.41–1.40)	0.46

c.1858 G>A	G	0.90	0.96	2.67 (1.47–4.85)	0.001	GG	85.0%	93.0%	Reference	
Asp 620 Asn	A	0.10	0.04			GA	10.5%	6.50%	GG versus GA: 1.77 (0.86–3.64)	0.11
						AA	4.5%	0.50%	GG versus AA: 9.85 (1.23–78.54)	0.008
									GG versus GA + AA: 2.34 (1.20–4.57)	0.011

c. 1921 G>A	G	0.87	0.94	2.34 (1.41–3.88)	0.001	GG	77.5%	89%	Reference	
Glu 641 Lys	A	0.13	0.06			GA	18.5%	11%	GG versus GA: 1.93 (1.09–3.41)	0.022
						AA	4.0%	0.0%	GG versus GA + AA: 2.35 (1.35–4.09)	0.002

c. 2002 G>A	G	0.93	0.94	1.18 (0.67–2.07)	0.56	GG	86.5%	88.5%	Reference	
Asp 668 Asn	A	0.07	0.06			GA	12.5%	11.5%	GG versus GA: 1.11 (0.61–2.03)	0.73
						AA	1.0%	0.0%		

c. 2173 A>C	A	0.54	0.63	1.45 (1.09–1.92)	0.01	AA	35%	41.0%	Reference	
Thr 725 Pro	C	0.46	0.37			AC	37.5%	44.0%	AA versus AC: 1.00 (0.64–1.56)	0.99
						CC	27.5%	15.0%	AA versus CC: 2.15 (1.24–3.71)	0.006
									AA + AC versus CC: 2.15 (1.31–3.53)	0.002

Chi-square test was used to compare the genotype and allele frequencies between cases and controls.

*P* value < 0.05 is considered to be statistically significant.

**Table 5:** Guaiacol and iodide oxidation activity of expressed human TPO protein.

Mutation	Guaiacol oxidation	Iodide oxidation
Wild type	0.76 ± 0.07	0.90 ± 0.05
Glu 641 Lys	ND	∗
Asp 668 Asn	0.63 ± 0.07	0.69 ± 0.27
Thr 725 Pro	ND	∗
Asp 620 Asn	ND	∗
Ser 398 Thr	0.70 ± 0.27	0.82 ± 0.29
Ala 373 Ser	0.62 ± 0.09	∗

Enzyme activity was activity expressed as Δ*A*·min^−1^·mg protein^−1^.

*Comparable with nonenzymatic reaction rate (0.51 ± 0.24). ND: no detectable activity (<10% of wild-type expressed TPO) [5].

## References

[1] Baker JR, Arscott P, Johnson J (1994). An analysis of the structure and antigenicity of different forms of human thyroid peroxidase. *Thyroid*.

[2] Banga JP, Mahadevan D, Barton GJ (1990). Prediction of domain organisation and secondary structure of thyroid peroxidase, a human autoantigen involved in destructive thyroiditis. *FEBS Letters*.

[3] Kimura S, Hong Y-S, Kotani T, Ohtaki S, Kikkawa F (1989). Structure of the human thyroid peroxidase gene: comparison and relationship to the human myeloperoxidase gene. *Biochemistry*.

[4] Rivolta CM, Esperante SA, Gruñeiro-Papendieck L (2003). Five novel inactivating mutations in the thyroid peroxidase gene responsible for congenital goiter and iodide organification defect. *Human mutation*.

[5] Bikker H, Baas F, De Vijlder JJ (1997). Molecular analysis of mutated thyroid peroxidase detected in patients with total iodide organification defects. *The Journal of Clinical Endocrinology and Metabolism*.

[6] Bakker B, Bikker H, Vulsma T, De Randamie JSE, Wiedijk BM, De Vijlder JJM (2000). Two decades of screening for congenital hypothyroidism in the Netherlands: TPO gene mutations in total iodide organification defects (an update). *Journal of Clinical Endocrinology and Metabolism*.

[7] Kotani T, Umeki K, Kawano J-I (2003). Partial iodide organification defect caused by a novel mutation of the thyroid peroxidase gene in three siblings. *Clinical Endocrinology*.

[8] Rodrigues C, Jorge P, Pires Soares J (2005). Mutation screening of the thyroid peroxidase gene in a cohort of 55 Portuguese patients with congenital hypothyroidism. *European Journal of Endocrinology*.

[9] Wu J-Y, Shu S-G, Yang C-F, Lee C-C, Tsai F-J (2002). Mutation analysis of thyroid peroxidase gene in Chinese patients with total iodide organification defect: identification of five novel mutations. *Journal of Endocrinology*.

[10] Maskari AM, Alnaqdy A (2006). Frequency of thyroid microsomal and thyroid peroxidase antibody levels in a selected group of Omani patients with Graves' Disease. *Kuwait Medical Journal*.

[11] Sandell EB, Kolthoff IM (1937). Micro determination of iodine by a catalytic method. *Mikrochimica Acta*.

[12] Pino S, Fang S-L, Braverman LE (1996). Ammonium persulfate: a safe alternative oxidizing reagent for measuring urinary iodine. *Clinical Chemistry*.

[13] Faam B, Daneshpour MS, Azizi F, Salehi M, Hedayati M (2012). Association between TPO gene polymorphisms and Anti-TPO level in Tehranian population: TLGS. *Gene*.

[14] Hedayati M, Jahromi MS, Yeganeh MZ, Daneshpour MS, Rad LH, Azizi F (2010). Association between serum level of anti-TPO titer and polymorphisms G1193/C Exon 8 and C2145/T Exon 12 of thyroid peroxidase gene in an Iranian population. *International Journal of Endocrinology and Metabolism*.

[15] Bikker H, Vulsma T, Baas F, De Vijlder JJM (1995). Identification of five novel inactivating mutations in the human thyroid peroxidase gene by denaturing gradient gel electrophoresis. *Human Mutation*.

[16] Huse M, Kuriyan J (2002). The conformational plasticity of protein kinases. *Cell*.

[17] Krupa A, Preethi G, Srinivasan N (2004). Structural modes of stabilization of permissive phosphorylation sites in protein kinases: distinct strategies in Ser/Thr and Tyr kinases. *Journal of Molecular Biology*.

